# Spatial organization of cutaneous microbiomes reveals putative microbial contributions to host chemical defenses in the American toad

**DOI:** 10.3389/fmicb.2026.1860796

**Published:** 2026-07-15

**Authors:** S. Donovan McCammon, Jeremy R. Chen See, Justin R. Wright, Samantha L. C. Anderson, Travis J. Russell, Regina M. Lamendella, Thomas J. Firneno

**Affiliations:** 1 Juniata College, Huntingdon, PA, United States; 2 Wright Labs, Huntingdon, PA, United States

**Keywords:** 16S rRNA gene, American toad (*Anaxyrus americanus*), chemical defenses, metagenomics, microbiome, mutualism

## Abstract

Chemical defenses are widely evolved throughout the tree of life. Animals can exploit mutualisms with toxin-producing symbionts as a mechanism of chemical defense. However, this has only begun to be explored in depth, and how these mutualisms may relate to how animals synthesize or acquire their toxins has been even less studied. True toads synthesize their own toxins and offer a novel system to study the interplay between the cutaneous skin microbiome and how it may contribute to toxin synthesis or biotransformation. In this study, we investigated whether the cutaneous microbiome of the American toad (*Anaxyrus americanus*) was spatially structured across body surfaces in relation to toxin storage and secretion and assessed whether microbial communities exhibit distinctive bacterial taxa involved in toxin-related biochemical pathways. To do this, we used 16S rRNA gene sequencing, diversity metrics, differential abundance comparisons, functional pathway predictions, and ecological interaction networks. Our results indicate that the dorsal and ventral cutaneous surfaces harbor distinct bacterial assemblages, with the dorsal surface being enriched for bacterial taxa associated with the predicted potential to degrade or transform structurally complex organic compounds. This study provides insights into how the toad skin microbiome may contribute to the chemical defenses of toads and could reveal novel aspects of host-microbiome interactions in amphibians.

## 1 Introduction

Chemical defenses are found in a wide range of organisms throughout the tree of life and have played a major role in the diversification and co-evolution of many taxa (Mebs, 2001; Dumbacher et al., 2000; Wortman-Wunder and Vivanco, 2011; Zhang, 2015). Although the chemical ecology of many animal toxins is increasingly well understood, the mechanisms underlying toxin structure, diversity, and evolution remain relatively unknown (Agrawal et al., 2012; Saporito et al., 2012; Ujvari et al., 2015; Rodríguez et al., 2017; Bókony et al., 2019). Animals that use chemical defenses can either biosynthesize them via genetic or metabolic mechanisms (Mebs, 2001; Agrawal et al., 2012; Siperstein et al., 1957; Cei et al., 1968; Speed et al., 2012; de Vasconcelos et al., 2022), sequester them from their environment (e.g., diet) (Dumbacher et al., 2000; Daly et al., 1994; Hutchinson et al., 2007; Gleibs and Mebs, 1999), or exploit mutualisms with toxin-producing symbionts (Kamalakkannan et al., 2017; Gil-Turnes and Fenical, 1992; Lindquist et al., 2005; Li et al., 2020). The latter mechanism has been identified as more common than originally thought, especially in marine organisms (Mebs, 2001; Gil-Turnes and Fenical, 1992; Lindquist et al., 2005; Li et al., 2020; Chau et al., 2013) but remains relatively unknown in terrestrial organisms. While microbes themselves can produce toxins that host organisms can employ for defense, the microbiome could also play a role in modifying toxins or toxin intermediates to aid the host’s toxin biosynthesis (Kamalakkannan et al., 2017; Chau et al., 2013). This may be especially true for organisms that use small metabolic toxins for defense and may lack all the chemical-modifying proteins required to carry a precursor molecule through the entire pathway to the toxin metabolite (Kamalakkannan et al., 2017; Gil-Turnes and Fenical, 1992; Lindquist et al., 2005; Li et al., 2020). Many studies of the relationship between microbes and chemical defenses focus on how the defensive toxins may affect the microbial communities (Chen et al., 2022; Raaymakers et al., 2017; Bulet and Stocklin, 2005; Campos et al., 2018; Kohl et al., 2014; Kohl and Dearing, 2016). However, aspects of how host-microbial symbioses may be shaped by toxin production and the role that they may play in the mediation of toxins is still a highly understudied aspect of chemical defense evolution (Caty et al., 2025).

Amphibians often exploit a range of chemical defenses, including toxic alkaloids (Fuhrman et al., 1969; Garraffo et al., 1993; Hanifin et al., 1999; Daly et al., 2005), cardiogenic steroids (Rodríguez et al., 2017; Siperstein et al., 1957), antimicrobial peptides (Raaymakers et al., 2017; Erspamer et al., 1985), and proteolytic enzymes (Jared et al., 2015), stored within and/or secreted from their skin. These toxins can be sequestered in various species (Saporito et al., 2012; Neuwirth et al., 1979) and biosynthesized in several others (Siperstein et al., 1957; Cei et al., 1968; de Vasconcelos et al., 2022). Research has also shown that specific microbial species associated with the amphibian skin microbiome may play a role in both toxin production and metabolism. For example, *Aeromonas*, *Pseudomonas*, *Shewanella*, and *Sphingopyxis* species cultured from the skin of *Taricha granulosa* (rough-skinned newts) produce tetrodotoxin under laboratory conditions (Vaelli et al., 2020). True toads (Anura: Bufonidae) offer a unique system for investigating the biosynthesis of their chemical defenses, which may include cardiac glycosides, alkaloids, and biogenic amines, with these chemicals primarily concentrated in the parotoid gland and dorsal skin (Hostetler and Cannon, 1974; Cannon and Hostetler, 1976). In addition to the host, the associated toad microbiome may have significant potential to influence toxin production and metabolism. Some microbes, including *Comamonas testosteroni*, *Acinetobacter johnsonii,* and *Flavobacterium* sp., can biotransform bufadienolides (cardiac glycosides), suggesting a mechanism for diversifying bufadienolide structures that could enhance their toxicity (Hayes et al., 2009). *Bacillus* sp. are present in the parotoid glands of cane toads and may play a role in mediating toxin biotransformation of secreted bufagenins and their derivatives across life stages (Kamalakkannan et al., 2017; Hayes et al., 2009). While studies indicate that these microbes may contribute to the production and/or degradation of amphibian toxins (Kamalakkannan et al., 2017; Vaelli et al., 2020), the specific metabolic pathways and microbial interactions within the toad skin microbiome remain underexplored, highlighting a promising area for future research on the ecological and coevolutionary relationships between toads and their associated microbiome.

Here, we aimed to determine how skin microbial communities differed with the location of toxin storage in the skin, and to reveal novel aspects of host-microbiome symbioses and microbial involvement in toxin biosynthesis, modification, or degradation. To do this, we performed 16S rRNA gene analysis on skin swabs collected from the dorsal and ventral surfaces of the American toad (*Anaxyrus americanus*) (1) compare dorsal and ventral bacterial communities and (2) identify bacteria and predict functional gene pathways that may be involved in toxin production. From prior literature on the toxin metabolites that bufonids synthesize, we know that they produce and possess steroid-derived cardiac glycosides (bufadienolides), tryptamine-related substances (bufotenins), and other metabolites that may make the toad distasteful. Therefore, we hypothesize that: (1) the skin microbial communities on the dorsal side (where toxins are secreted and stored) and ventral side (where toxins are not stored and/or secreted) of the toad will differ due the their abilities to protect themselves from the toxins the toad produces through degradation pathways; and (2) there will be bacterial taxa on the dorsal side of the toads capable of the mediation, metabolism, or biosynthesis of toxin metabolites (e.g., propanoates, glucosinolates, terpenoids, steroids) that the toads can use as intermediates for their own toxin production or that the toads co-opt for defense.

## 2 Materials and methods

### 2.1 Sample collection

We collected 80 American toads (*A. americanus*) by hand from small ponds or roads in Central Pennsylvania in Summer 2024. We captured individuals using fresh nitrile gloves, sexed them, and then rinsed their dorsal and ventral surfaces with ~100 mL of sterile water before sampling to eliminate transient bacteria (i.e., those present in the environment but not otherwise associated with amphibian skin). We swabbed the toads in duplicate on the dorsal and ventral sides separately (4 swabs per individual; *n* = 320 total) using 10 strokes per swab. Individual swabs were placed into a sterile 1.5 mL tube containing 150 μL of Zymo DNA/RNA Shield (Zymo Research, Irvine, CA, United States) and then placed on ice for the duration of sampling and transferred to a − 80 °C freezer for storage until DNA extraction.

### 2.2 Molecular methods

We extracted DNA by combining duplicate swabs for single extractions (*n* = 160) using a ZymoBIOMICS DNA/RNA Miniprep Kit (Zymo Research, Irvine, CA, United States) according to the manufacturer’s protocol, and eluted the extractions with 50 μL of DNase/RNase-free water. We quantified DNA in all extracts using an Invitrogen Qubit 4 Fluorometer and 1X Qubit dsDNA High Sensitivity Assay Kit (Thermo Fisher Scientific, Waltham, MA, USA).

We performed 16S rRNA Illumina-tag PCR reactions on all 160 DNA extracts per the Earth Microbiome Project’s protocol (Walters et al., 2015), and a negative control (PCR-grade nuclease-free water) was processed in parallel with the samples. We pooled PCR products in equimolar concentrations and gel-purified them on a 2% agarose gel using the QIAquick Gel Purification Kit (Qiagen, Frederick, Maryland, USA). Before sequencing, we quality-checked the purified pools using an Agilent 2100 BioAnalyzer and High Sensitivity DNA kit (Agilent Technologies, Santa Clara, California, USA), and subsequently sequenced them using Illumina MiSeq v2 kits to produce paired-end 250-base-pair reads.

### 2.3 Bioinformatic and statistical analysis

We used QIIME2 (Bolyen et al., 2019) to process and analyze our raw sequence data. We determined the initial quality, Phred Q scores, and cumulative expected error for each position. Using the DADA2 pipeline (Callahan et al., 2016) within QIIME2, we filtered our data based on quality by truncating the forward reads at a length of 195 base pairs (bp) and reverse reads at a length of 183 bp with an expected error for both of 0.5. We then merged forward and reverse reads, removed chimeric sequences, and assigned the remaining reads as amplicon sequence variants (ASVs).

We determined taxonomic information for our ASVs using a pre-trained Naive Bayes classifier in QIIME2, with a database of 515F/806R sequences based on SILVA 138.2 (Chuvochina et al., 2026). Additionally, we used MAFFT (Katoh and Standley, 2013) and FastTree2 (Price et al., 2010) through QIIME2 to generate a rooted phylogenetic tree. To prevent non-bacterial signals from influencing results, ASVs identified as mitochondrial or chloroplast were removed, as they likely represent eukaryotic contamination. After filtration, we removed samples with fewer than 1,000 sequences remaining from the ASV table.

To understand the bacterial diversity within samples, we calculated within-sample diversity (α-diversity) by subsampling the ASV table at 10 depths between 100 and 1,000 for the Shannon measures for Richness and Evenness (Shannon, 1948), Faith’s Phylogenetic Diversity (Faith, 1992), Observed Features, and Pielou’s Evenness metrics (Pielou, 1966). Good’s coverage (Good, 1953) with the same rarefaction settings was used to evaluate coverage at the maximum rarefaction depth. We then compared alpha diversity metrics between dorsal and ventral swabs using a Kruskal-Wallis test (*p* < 0.05). To understand bacterial diversity between samples (β-diversity), we first mitigated differences due to sequencing depth by applying cumulative sum scaling normalization to our ASV table (Paulson et al., 2013). We then calculated distances using the weighted UniFrac metric based on our normalized table and phylogenetic tree (Lozupone et al., 2007). We visualized the weighted UniFrac distance matrix as a Principal Coordinates Analysis (PCoA) plot using the qiime2r package to import it into R and then the vegan package to perform PCoA ordination. We used the PERMANOVA (Adonis) test to determine significance between dorsal and ventral swabs and between sampling locations.

We used Linear Discriminant Analysis Effect Size (LEfSe) (Segata et al., 2011) to determine significant differences in abundances of taxa on the dorsal and ventral sides (Linear Discriminant Analysis (LDA) score (log_10_) ≥ 2.5 and *p* < 0.05). We ran PICRUSt2 (Douglas et al., 2020) on our filtered ASV table to determine predictive gene pathways, followed by LEfSe to identify which pathways were predicted to differ between dorsal and ventral sides. To complement our LEfSe analysis and account for covariates, we used MaAsLin3 (Mallick et al., 2021) at the species level with default settings, except small_random_effects set to TRUE, to identify differential features while accounting for repeated measures by including toad IDs as a random effect in the model.

We also used the Co-Occurrence Network (CoNet; Faust and Raes, 2016) tool in Cytoscape (Shannon et al., 2003) to assess possible interactions among ASVs present in more than 50% of our samples, using Pearson and Spearman correlations. Prior to network analysis, ASV counts were collapsed to the species level, meaning all taxonomic features had seven levels, though many could not be identified as a specific species and were thus given an ambiguous classification at the species level, before conversion to relative abundances. The corresponding *p*-values were combined using CoNet with the Brown method (Brown, 1975), and a Benjamini-Hochberg correction (Benjamini and Hochberg, 1995) was applied before the resulting corrected *p*-values were assessed for significance (α = 0.05), with only significant correlations retained.

## 3 Results

### 3.1 Sequencing assessment and statistics

In total, 4,633,492 raw sequences were generated across 160 sequenced samples. Following quality filtering and chimera removal, 144 samples had sufficient depth (≥1,000 remaining sequences) to be included for analysis. We removed dorsal (*n* = 9) and ventral (*n* = 7) swabs that had low depth and excluded them from further processing. Our final dataset included the remaining 144 samples (*n* = 71 dorsal; *n* = 73 ventral), with a total of 2,807,417 filtered sequences. Filtered sequence counts ranged from 1,024 to 70,266 within the retained samples, with an average of 19,361 sequences.

### 3.2 Alpha and beta diversity of toad cutaneous microbiomes

**Figure 1:**
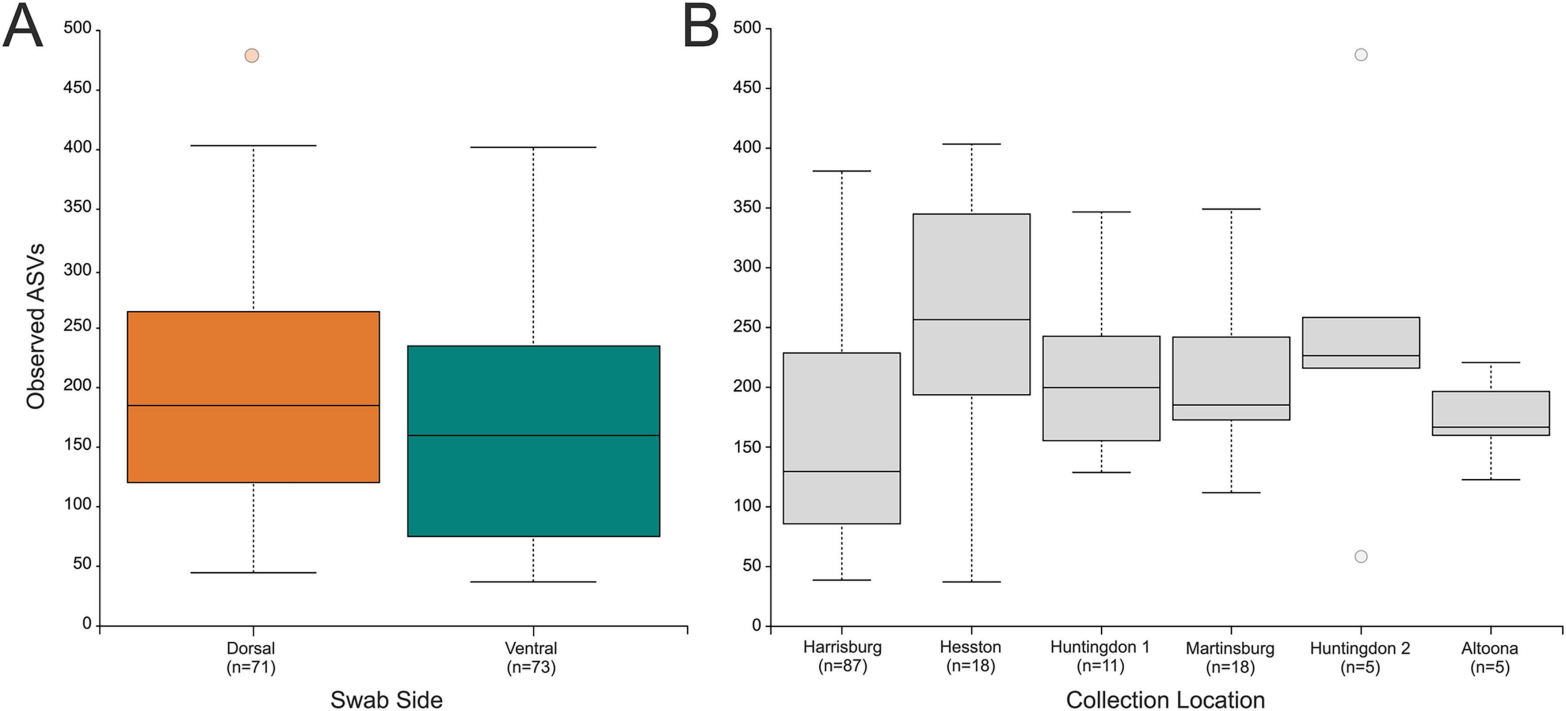
**(A)** Alpha diversity comparisons of bacterial ASVs on dorsal versus ventral cutaneous swabs. **(B)** Alpha diversity comparisons of bacterial ASVs by sampling location. Observed ASVs in dorsal and ventral skin swabs from toads. Boxplots display the distribution of alpha diversity (observed ASVs) across sampling sites. Points beyond the whiskers denote statistical outliers. This comparison highlights site-specific variation in cutaneous microbial richness. There is no statistical difference in ASV richness between dorsal and ventral swabs (Kruskal-Wallis, *p* = 0.058). There is a statistical difference in ASV richness between sampling sites (Kruskal-Wallis, *p* = 0.002). When pairwise comparisons among locations were evaluated using Kruskal-Wallis tests, the following were significant: Harrisburg vs. Hesston (*p* = 0.002), Harrisburg vs. Huntingdon 1 (*p* = 0.033), Harrisburg vs. Martinsburg (*p* = 0.008).

**Figure 2:**
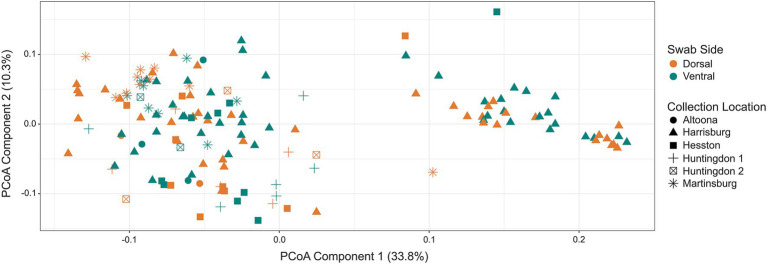
Principal coordinates analysis (PCoA) plot of samples remaining after all quality filtering (*n* = 144) based on the weighted UniFrac distance matrix calculated with QIIME2. Location and swab side together explain a significant amount of variation among the samples (PERMANOVA, *n* = 999 permutations, *p* = 0.001). Location alone showed significant clustering (PERMANOVA, *p* = 0.001). However, samples did not cluster significantly by swab side (PERMANOVA, *p* = 0.191).

Analysis of ASV richness (α-diversity) revealed that the toad cutaneous microbiome harbors a bacterial richness ranging from 36–479 ASVs across both dorsal (average ASVs = 198) and ventral skin surfaces (average ASVs = 170) (Figure 1A). Rarefaction curves with Good’s coverage confirmed 1,000 sequences were sufficient depth to capture most of the expected diversity in the majority of samples (Supplementary Figure S1). The number of observed ASVs ranged broadly from fewer than 50 to over 400 per swab. When comparing dorsal and ventral swabs with Kruskal-Wallis, no significant differences in Observed ASVs (*p* = 0.058), Faith’s Phylogenetic Diversity (*p* = 0.125), Pielou’s evenness (*p* = 0.139), or Shannon’s Index (*p* = 0.057) were detected, indicating that both surfaces support similarly diverse bacterial assemblages (Figure 1; Supplementary Figures S2–S4). In contrast, α-diversity varied significantly across sampling sites for Observed Features (*p* = 0.002), Faith’s Phylogenetic Diversity (*p* < 0.001), Pielou’s evenness (*p* < 0.001), and Shannon’s Index (*p* < 0.001) (Figure 1; Supplementary Figures S2, S4). β-diversity analysis revealed that both dorsal and ventral skin surfaces harbor distinct microbial communities (Figure 2), though the differences in community composition between surfaces were not statistically significant (PERMANOVA, *n* = 999 permutations; *p* = 0.191). Contrastingly, site-level comparisons demonstrated significant differences in microbial community structure across sampling locations (PERMANOVA, *p* = 0.001).

### 3.3 Differentially abundant bacterial taxa

**Figure 3:**
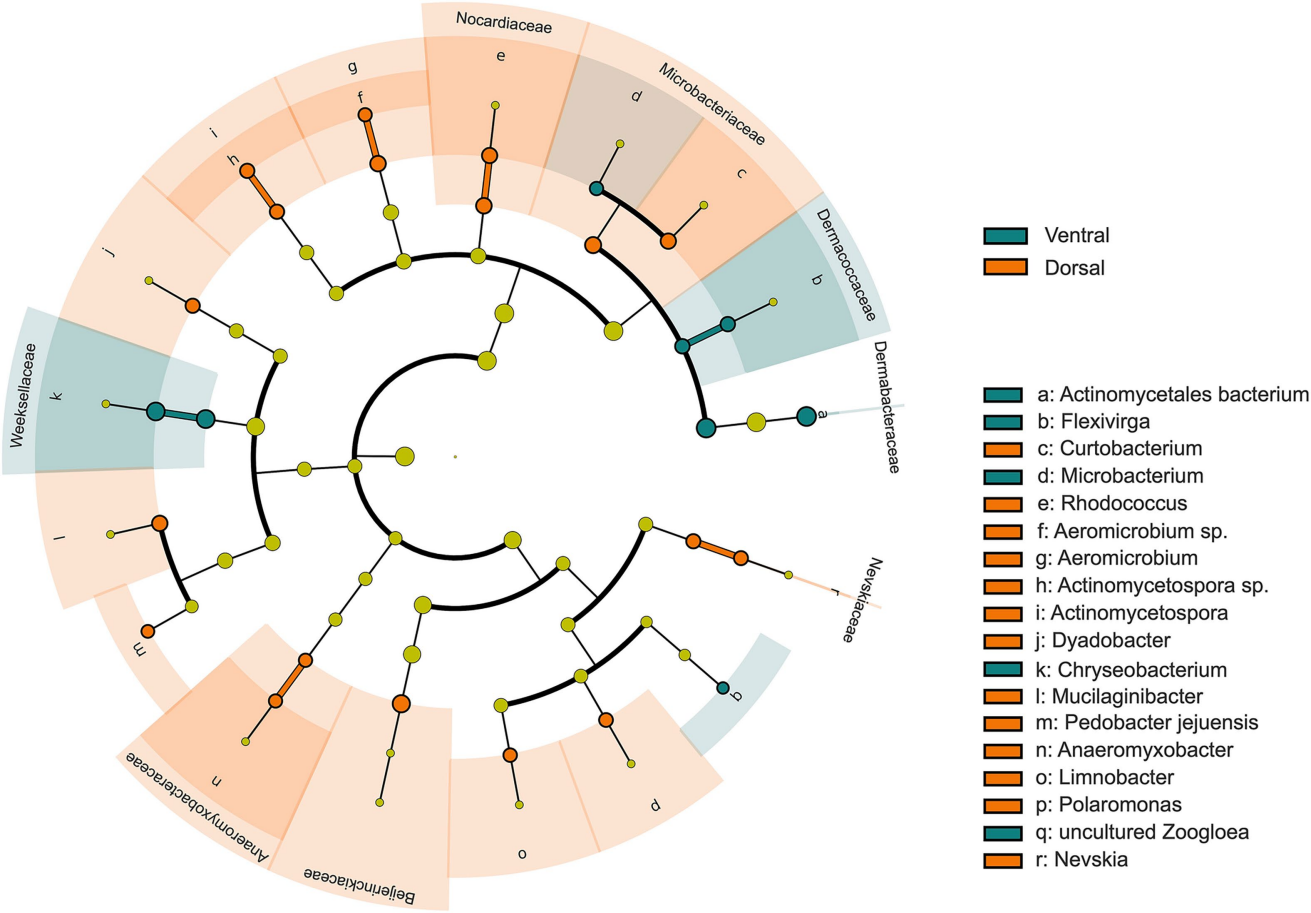
LEfSe cladogram of bacterial taxa differentially abundant between dorsal (orange) and ventral (teal) cutaneous swabs from toads. Taxa are organized by phylogenetic hierarchy, with circles representing nodes from phylum to genus level. Colors indicate the group in which each taxon is significantly enriched, with shaded regions highlighting enriched clades. A Linear Discriminant Analysis (LDA) score threshold of 2.5 was applied to identify differentially abundant features with at least family level taxonomy identified. This analysis demonstrates distinct microbial signatures associated with dorsal versus ventral skin microhabitats.

Differential taxa abundance analysis was able to reveal statistically significant differences in bacterial composition between dorsal and ventral cutaneous microbiomes of the American toad (Figure 3), though species-level resolution was limited within our samples (Supplementary Figure S5). Differential abundance analyses revealed that dorsal skin microbiomes were significantly enriched in 24 different bacterial taxa, while ventral microbiomes were only enriched in 12 different taxa (Supplementary Figure S6; Supplementary Table S1). Dorsal microbiomes were enriched in Actinobacteria-dominated taxa, including *Rhodococcus*, *Curtobacterium*, *Aeromicrobium*, and *Actinomycetospora*, as well as select Proteobacteria such as *Allorhizobium* (Rhizobiaceae), *Polaromonas* (Comamonadaceae), *Limnobacter* (Burkholderiaceae), *Nevskia* (Nevskiaceae) and members of the family Beijerinckiaceae (Figure 3). Ventral-associated taxa belonged primarily to the Actinobacteria families including, Dermabacteraceae, Dermacoccaceae (*Flexivirga*) and Microbacteriaceae (*Microbacterium*), as well as the Bacteroidota family Weeksellaceae (*Chryseobacterium*) (Figure 3). MaAsLin analysis (Supplementary Figure S3) showed strong concordance in the identification of differentially abundant taxa between dorsal and ventral skin. Both LEfSe and MaAsLin analyses highlighted a consistent enrichment of several different Actinobacteria on the dorsal surface, with Dermacoccaceae and Dermabacteraceae being enriched in ventral communities (Supplementary Figure S7). MaAsLin analysis additionally identified *Roseburia*, *Parabacteroides*, and an uncultured member of the Ruminococcaceae family as enriched in dorsal skin microbiomes.

Differential abundance analysis revealed distinct site-specific bacterial signatures across the six sampling locations. Martinsburg toad microbiomes showed significant enrichment of several Sphingobacteria and members of the Intrasporangiaceae. Samples collected from Huntingdon were characterized by elevated abundances of multiple Alpha- and Betaproteobacteria, whereas Harrisburg specimens were enriched in diverse Gammaproteobacteria. Altoona toad microbiomes exhibited increased relative abundances of several Actinobacteria, while Hesston samples displayed enrichment of taxa within the Xanthomonadales. Collectively, the LEfSe analysis demonstrated that each geographic site possessed a unique set of differentially abundant bacterial taxa (Supplementary Figure S8).

### 3.4 Differentially abundant functional predictions

**Figure 4:**
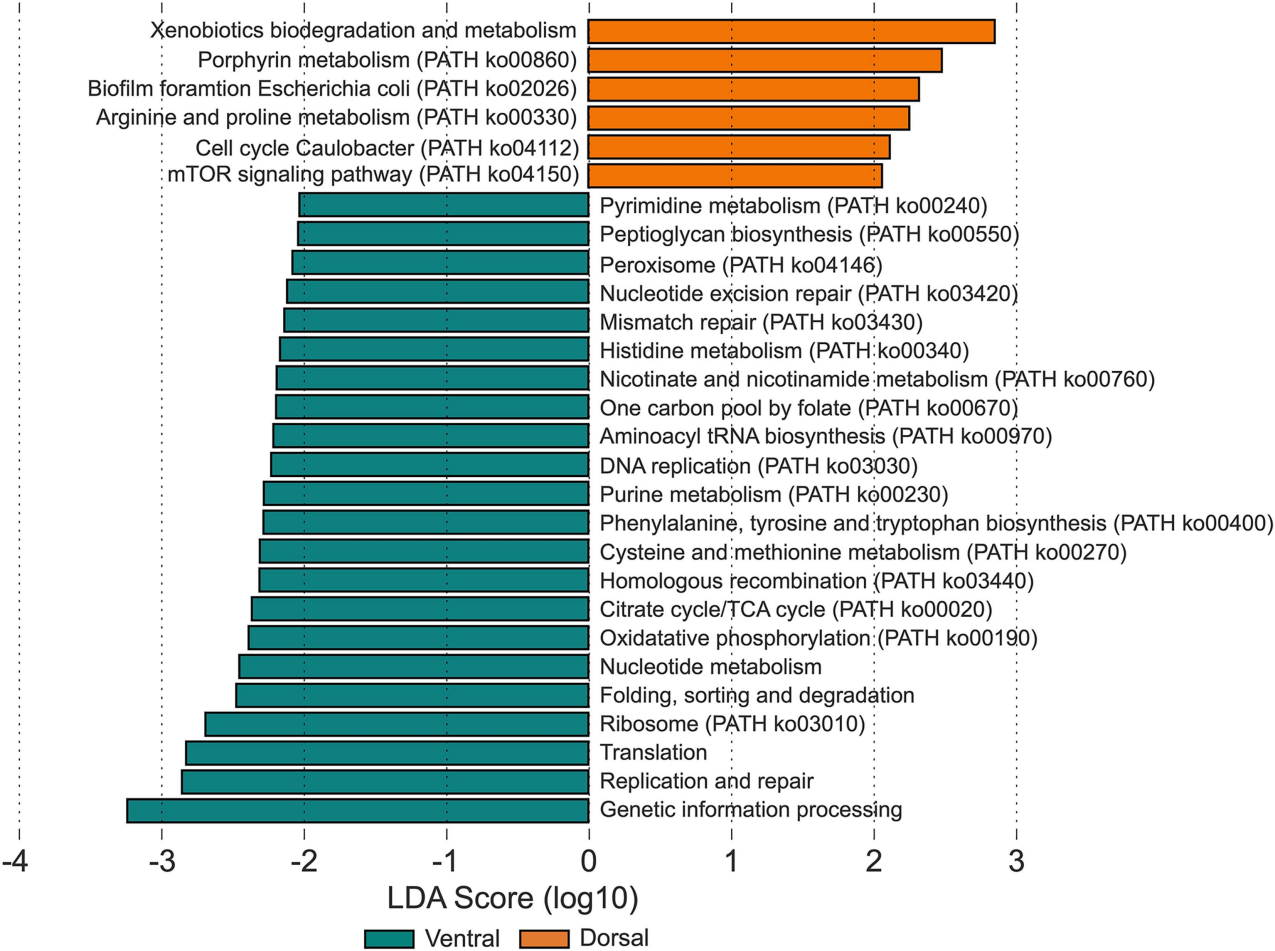
LEfSe analysis of PICRUSt2-predicted functional gene pathways enriched in dorsal (orange) and ventral (teal) cutaneous swabs. Horizontal bars indicate differentially abundant KEGG pathways with their corresponding LDA scores (log10), where positive scores denote enrichment in dorsal cutaneous swabs and negative scores denote enrichment in ventral cutaneous swabs. Functions enriched in the dorsal microbiome include xenobiotic biodegradation and metabolism, and porphyrin metabolism, while ventral microbiomes demonstrate enrichment in genetic information processing and replication and repair. An LDA score (log10) threshold of 2.0 was applied.

Predictive functional profiling using PICRUSt2 revealed differences in inferred metabolic potential between dorsal and ventral cutaneous microbiomes (Figure 4). We emphasize that PICRUSt2 generates predictions based on phylogenetic inference from 16S rRNA gene data and does not directly measure gene content or functional activity. Within this framework, dorsal-associated microbiomes were predicted to be enriched in pathways related to xenobiotic biodegradation and metabolism, porphyrin metabolism, arginine and proline metabolism, and biofilm formation. These predicted pathways align with the taxonomic enrichment of Proteobacteria and Actinobacteria on dorsal surfaces. Taxonomic contribution analysis indicated that Proteobacteria, including *Pseudomonas* and *Sphingomonas*, contributed substantially to predicted xenobiotic metabolism pathways (Supplementary Table S3). Ventral microbiomes were predicted to be enriched in pathways associated with cellular maintenance and growth, including replication, repair, and translation (Figure 4).

### 3.5 Ecological networks of cutaneous toad microbiomes

**Figure 5:**
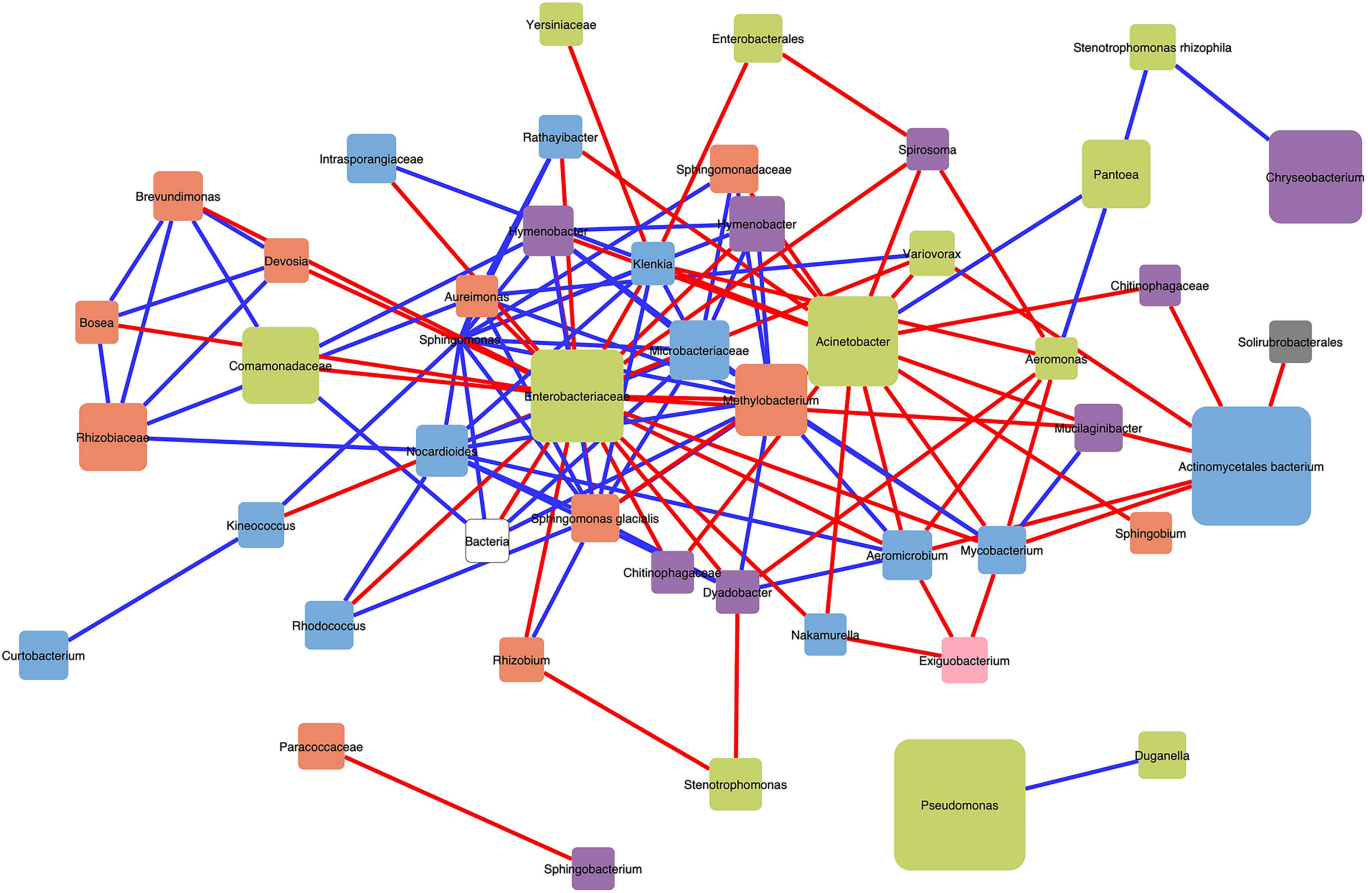
Co-occurrence network of bacterial taxa identified from 16S rRNA gene sequencing of dorsal toad cutaneous swabs, constructed using CoNet. Nodes represent bacterial genera, with node color denoting Class level identification (Actinobacteria = blue, Alphaproteobacteria = peach, Bacilli = pink, Bacteroidia = purple, Gammaproteobacteria = olive, Thermoleophilia = dark grey, Verrucomicrobiae = light gray, unspecified to Class level = white) and node size proportional to relative abundance. Edges indicate significant co-occurrence (blue, positive correlations) or co-exclusion (red, negative correlations) relationships, as determined by Bray-Curtis, Kullback-Liebler Distances, and Spearman correlations (Benjamini-Hochberg adjusted merged *p* ≤ 0.05). The network structure highlights distinctive interaction patterns in the dorsal bacterial community, suggesting habitat-specific bacterial associations and potential ecological interactions shaping the different bacterial assemblages that exist in dorsal toad skin microbiomes.

Co-occurrence network analysis revealed that several bacterial taxa enriched in dorsal microbiomes were significant central nodes within the microbiome networks (Figure 5). The dorsal network was dense and highly connected, dominated by Actinobacteria, Bacilli, and Proteobacteria. Most Actinobacterial taxa in the network showed positive correlations with other actinobacterial taxa and occupied central hub positions within the dorsal microbiome network. Bacilli taxa also nearly exclusively shared positive, co-occurring relationships with other bacterial taxa in the network. Several proteobacterial taxa had negative correlations with other members of the dorsal bacterial community.

## 4 Discussion

The goal of this study was to investigate whether the cutaneous microbiome of the American toad (*A. americanus*) is spatially structured across body surfaces (dorsal versus ventral) in relation to toxin storage and secretion, and to assess whether dorsal-associated microbial communities exhibit distinctive bacterial taxa involved in toxin-related biochemical pathways. We expected to see differences in the bacterial communities on the cutaneous surfaces where toads store/secrete toxins (dorsal) versus where they do not (ventral), and, further, that the community on the dorsal cutaneous surfaces would harbor bacterial species capable of metabolizing or biosynthesizing toxin metabolites. We used 16S rRNA gene sequencing, differential abundance testing, functional gene prediction, and bacterial network analysis, to identify bacterial taxa and predictive metabolic capacities enriched on toxin-secreting skin surfaces. This work provides a foundation for understanding how host-associated bacteria may be involved in amphibian chemical defense systems and addresses the potential contributions of microbe-mediated metabolism to toxin biosynthesis and transformation, as well as to ecological diversification in bufonid toad chemical defenses.

### 4.1 Spatial structuring of the toad cutaneous microbiome

Our 16S rRNA gene data have provided novel evidence that the cutaneous microbiome of the American toad is spatially structured. While alpha and beta diversity metrics showed similar overall richness and evenness by body site, differential abundance analyses indicated that dorsal and ventral surfaces harbor distinct bacterial assemblages. These results align with previous work in fire-bellied toads, which demonstrated spatial structuring in amphibian skin microbiomes, with different microhabitats imposing distinctive selective filters on microbial communities (Bataille et al., 2016). Differential abundance analysis revealed that dorsal skin communities harbored enriched bacterial assemblages dominated by Actinobacteria (e.g., *Rhodococcus*, *Aeromicrobium, Actinomycetospora*, *Curtobacterium*, and Nocardiaceae). Differential taxonomic composition between the dorsal and ventral cutaneous microbiomes demonstrates the extent to which microhabitat structure and physiochemical conditions shape amphibian skin-associated microbial communities (Abarca et al., 2018; Kueneman et al., 2014; Santos et al., 2023). The significant enrichment of Actinobacteria, including Microbacteriaceae and Nocardiaceae, on the dorsal surface reflects a microbiome dominated by bacterial assemblages known for their resilience to desiccation, UV exposure, and metabolism of complex organic compounds (Bataille et al., 2016; Barka et al., 2015; Mohammadipanah and Wink, 2016). Furthermore, these enriched bacteria formed highly connected, positively co-occurring networks exclusively in dorsal microbiomes. The function of these bacterial assemblages is also not entirely surprising with relation to the dorsal skin surface of toads as this surface is not only the location of toxin producing glands but also is often more prone to desiccation/drying out and UV exposure than the ventral surface. Together, these findings suggest that the dorsal skin exerts a nonrandom and different selective pressures on bacterial membership as compared to ventral bufonid cutaneous microbiomes.

Despite clear differences in relative abundance between dorsal and ventral communities, our beta diversity analyses indicated that site-level environmental variation exerted a stronger influence on microbiome structure than body region. Differences in local habitat characteristics, including soil type, moisture, and vegetation, likely shape the microbial source pool from which toad cutaneous microbiomes are assembled (Kueneman et al., 2014; Douglas et al., 2021; Estrada et al., 2019; Albecker et al., 2019; Kueneman et al., 2019). This pattern is consistent with previous work showing that the habitat and population characteristics often explain more variation in amphibian skin microbiomes than host phylogeny alone (Abarca et al., 2018; Garcia-Recinos et al., 2019). These findings suggest that while dorsal and ventral surfaces represent distinct microhabitats, they are colonized and filtered from a regional species pool shaped by local environmental conditions. Ultimately, our results indicate that site-specific environmental conditions populate toads with available symbionts present, while body region fine-tunes/selects subsets of that pool that are maintained on the host.

### 4.2 Dorsal actinobacteria and proteobacteria and putative toxin-related functions

The strongest spatial differentiation observed in this study was the enrichment of phylogenetically clustered Actinobacteria/Actinomycetota and Proteobacteria/Pseudomonadota on dorsal skin surfaces. Actinobacteria are recognized for their metabolic versatility and prolific production of secondary metabolites, including antibiotics, antifungals, and cytotoxic compounds (Barka et al., 2015; Mohammadipanah and Wink, 2016; Jose et al., 2021). The dominance of closely related actinobacterial lineages on dorsal skin suggests non-random phylogenetic clustering driven by shared functional traits. Several Actinobacteria genera known for their capacity to transform structurally complex and toxic compounds were enriched in dorsal toad microbiomes, including *Rhodococcus* (dorsal average ( $\bar{D}$ ) = 0.682%, ventral average ( $\bar{V}$ ) = 0.270%)*, Curtobacterium* ( $\bar{D}$ = 0.732%, $\bar{V}$ = 0.475%)*, Aeromicrobium* ( $\bar{D}$ = 0.884%, $\bar{V}$ = 0.352%)*, and Actinomyceteospora* ( $\bar{D}$ = 0.302%, $\bar{V}$ = 0.094%). For example, *Rhodococcus* species perform xenobiotic metabolism, including degradation of aromatic hydrocarbons, steroids, pesticides, and antibiotics (Larkin et al., 2005; Martínková et al., 2009), and are resilient to desiccation and UV exposure. *Nocardia* ( $\bar{D}$ = 0.036%, $\bar{V}$ = 0.008%) species possess the biosynthetic capacity to produce antimicrobial and cytotoxic secondary metabolites and can degrade complex xenobiotics (Luo et al., 2014; Nonthakaew et al., 2025). In addition, *Rhodococcus* and *Curtobacterium* taxa have been shown to transform phenolic compounds, nitroaromatic pollutants, and other organic toxins (Ueno et al., 1983; Levy-Booth et al., 2019; Khleifat et al., 2022; Hu et al., 2023). The enrichment of *Actinomycetospora* in dorsal cutaneous microbiomes is notable given its affiliation with the Pseudonocardiaceae, a family of actinomycetes known for complex secondary metabolism and the biotransformation of organic compounds (Abarca et al., 2018; Jiang et al., 2008; van der Meij et al., 2017). Although direct toxin production by *Actinomycetospora* has not been demonstrated in amphibian systems, its association with the environmentally exposed dorsal skin suggests a potential role in chemical mediation through secondary metabolite production or modification of host- or environment-derived compounds (Walke et al., 2014). However, because parotid glands were not directly sampled here, these associations reflect patterns on the external skin surface and cannot be directly extended to microbial processes occurring within toxin-producing glandular tissues.

The enrichment of Proteobacteria on dorsal skin, including members of the Comamonadaceae (*Polaromonas*; $\bar{D}$ = 0.185%, $\bar{V}$ = 0.133%), Burkholderiaceae (*Limnobacter*; $\bar{D}$ = 0.238%, $\bar{V}$ = 0.050%), Nevskiaceae (*Nevskia*; $\bar{D}$ = 0.224%, $\bar{V}$ = 0.020%), and Beijerinckiaceae; ( $\bar{D}$ = 3.834%, $\bar{V}$ = 2.541%), reflects the prevalence of environmentally derived, metabolically versatile taxa adapted to exposed surface habitats. Many of these lineages are associated with aerobic metabolism, stress tolerance, and the transformation of diverse organic substrates, including plant- and soil-derived compounds, and have been reported from amphibian skin where environmental filtering favors flexible, surface-adapted microbes (Walke et al., 2014; Bletz et al., 2017). The presence of these Proteobacteria on dorsal skin may therefore reflect both frequent environmental contact and selective pressures favoring taxa capable of competing in chemically complex, microbially antagonistic cutaneous environments (Jiménez et al., 2019).

Predicted functional profiling using PICRUSt2 suggested that dorsal communities were enriched in inferred pathways related to xenobiotic biodegradation and metabolism, porphyrin metabolism, and amino acid metabolism (Figure 4). These predicted functions align with the known metabolic capacities of dominant Proteobacteria and Actinobacteria. While PICRUSt2 provides inferred rather than direct functional measurements, the convergence of phylogenetic clustering, pathway enrichment, and known metabolic traits suggests that dorsal-associated taxa have the capacity to be involved in the transformation or modification of host- or environment-derived compounds, such as bufadienolides, though this cannot be directly confirmed here. Proteobacterial genera such as *Pseudomonas* ( $\bar{D}$ = 7.080%, $\bar{V}$ = 6.70%) and *Sphingomonas* ( $\bar{D}$ = 8.334%, $\bar{V}$ = 7.137%) contributed most strongly to predictions of xenobiotic metabolism (Supplementary Table S3) and are well known for transforming diverse organic compounds on biological surfaces (Stolz, 2009; Palleroni, 2010; Rodriguez et al., 2020; Russell et al., 2021). Actinobacteria, similarly enriched on dorsal skin, are widely recognized for their roles in secondary metabolism and defensive symbioses across animal systems (Brucker et al., 2008; Engl et al., 2018). However, it should be noted that no specific genes related to toxin mediation/biosynthesis of known toad toxin compounds were found and that these interpretations remain predictive, and future multi-omics approaches will be necessary to resolve these functional roles directly.

### 4.3 Ecological network structure and defensive symbiosis and evolution

Co-occurrence network analysis revealed that the dorsal cutaneous microbiome of the American toad forms a highly interconnected ecological network, with Actinobacteria, Bacilli, and Proteobacteria dominating the network backbone. Actinobacterial taxa occupied central hub positions and exhibited mostly positive correlations with one another in the cooccurrence network. These patterns are consistent with potential cooperative functionally complimentary relationships, but could also reflect shared environmental preferences, synchronous colonization dynamics, or neutral assembly processes. Actinobacteria are well known for their roles in secondary metabolite production, stress tolerance, and antimicrobial activity. Therefore, their centrality within the dorsal network is consistent with the need for defensive, metabolically versatile taxa on skin surfaces subject to UV radiation, desiccation, and other environmental challenges (Barka et al., 2015; Jose et al., 2021; Kaltenpoth, 2009; Lee et al., 2020). Similar network architectures have been described in defensive symbioses across diverse systems, such as insects, where actinobacterial symbionts occupy central network positions and provide host protection through the production of antibiotics and other bioactive compounds (Engl et al., 2018; Kaltenpoth, 2009; Kaltenpoth et al., 2005). Bacilli similarly displayed largely positive co-occurrence patterns, indicating a shared ecological niche and supporting network cohesion. Bacilli have previously been shown to be involved in the biotransformation of bufadienolides (Kamalakkannan et al., 2017). In contrast, several proteobacterial taxa exhibited negative correlations with other members of the dorsal community, suggesting competitive interactions or niche exclusion (Walke et al., 2014; Kueneman et al., 2016). Altogether, the network modeling of the dorsal cutaneous microbiome is a highly structured ecological system in which Actinobacteria and Bacilli act as central hub nodes. At the same time, negative interactions involving Proteobacteria may contribute to a functional balance on the dorsal skin surface.

Bufonid toads synthesize and store bufadienolides and other bioactive compounds in specialized skin glands, and their skin secretions exhibit antimicrobial and anti-predator activities (Garg et al., 2007; Kowalski et al., 2018). Emerging evidence in toads and other amphibians (Caty et al., 2025) indicates a reciprocal relationship, whereby microbial processes influence these host chemical defenses, and the host chemical defenses shape the composition and functions of microbial communities. In *Bufo bufo* tadpoles, bufadienolide profiles shift with environmental bacterial community structure, suggesting microbial mediation of toxin composition (Bókony et al., 2016), while in cane toads (*Rhinella marina*), parotoid-associated bacteria can biotransform toxins, contributing to toxin diversity across tissues and developmental stages (Kamalakkannan et al., 2017; Hayes et al., 2009). Together, these findings support the idea that host physiology, microbiomes, and environment can shape defensive phenotypes. Our results demonstrate that dorsal skin, where bufadienolide-rich secretions are released, harbors an Actinobacteria-enriched microbial community with predicted xenobiotic and secondary metabolic pathways. Though no genes directly involved in the mediation or biosynthesis of known toxin compounds were found, these dorsal assemblages may contribute to chemical defense evolution via toxin tolerance or transformation, and/or the biosynthesis of microbial secondary metabolites. While direct functional validation is needed, our findings suggest that bufonid chemical defenses may arise not only from host biosynthesis, but from interactions among the host, microbiome, and environment.

### 4.4 Limitations and future directions

While this study provides new insight into the spatial structuring of the cutaneous microbiome of the American toad and its potential association with toxin-related processes, some limitations should be acknowledged. First, our analyses relied on 16S rRNA gene sequencing, which has limited taxonomic resolution and only allows for predictive functional capacity. Although PICRUSt2-based functional predictions, combined with phylogenetic clustering and network structure, suggest enrichment of toxin-relevant metabolic pathways in dorsal skin, these predictions do not confirm the presence, expression, or metabolic activity of genes. Furthermore, these predictive analyses did not directly target the specific compound classes (e.g., propanoates, glucosinolates, terpenoids, steroids) that we originally hypothesized. Future work integrating shotgun metagenomics and metatranscriptomics from matched DNA and RNA extracts, as well as direct culture-based assays and metabolic tests are necessary to more directly identify microbial genes, pathways, and transcriptional activity involved in toxin synthesis, modification, or degradation. Second, our sampling focused on external dorsal and ventral skin surfaces and did not include the parotoid glands, which are the primary sites of toxin storage and potential synthesis in bufonid toads. As a result, while dorsal skin represents a relevant interface for toxin secretion, we cannot directly link the identified microbial taxa to toxin biosynthesis or modification within glandular tissues. Future studies that integrate targeted sampling of parotoid glands with multi-omics approaches will be essential for directly evaluating microbial involvement in toxin production and transformation. Finally, our understanding of the biosynthetic pathways of specific toxin metabolites in bufonid toads is extremely limited. Without knowing the metabolic intermediates and chemical-modifying enzymes involved in these pathways, it is difficult to determine the specific role that cutaneous microbial communities play in the biotransformation of these toxins. However, this is becoming more accessible as genomic resources for both the microbiomes of these host organisms and the host organisms themselves are generated and accumulated. While our study reveals strong associations among microbial taxa, predicted functions, and skin regions, future studies will allow a more mechanistic understanding of how host–microbiome interactions contribute to amphibian chemical defenses and their evolution.

## Data Availability

The datasets presented in this study can be found in online repositories. The names of the repository/repositories and accession number(s) can be found at: https://www.ncbi.nlm.nih.gov/, PRJNA1397191.
