# Supplementary material for: Spatial organization of cutaneous microbiomes reveals putative microbial contributions to host chemical defenses in the American toad
Source: Front Microbiol. 2026 Jul 15;17:1860796. doi: 10.3389/fmicb.2026.1860796 (PMC13416787; doi:10.3389/fmicb.2026.1860796)
Supplement: Supplementary file 1 [file Table_1.DOCX]

**Supplementary Materials**


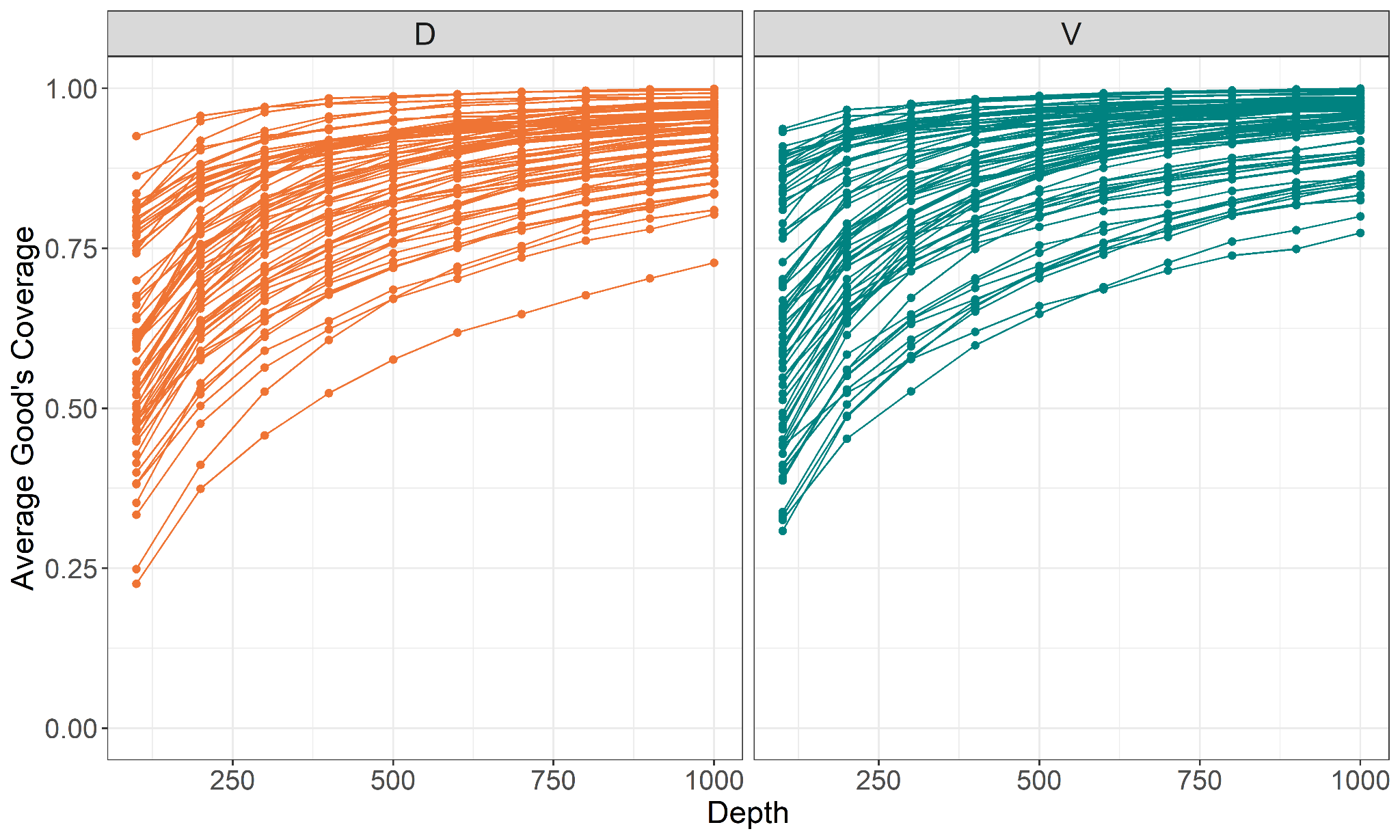


**Figure S1.** Average Good’s coverage values per sample for the depths used in rarefactions. Most samples yielded values close to 1 and approached horizontal asymptotes as rarefaction depth approached 1000 sequences. Specifically, 108 of the 144 samples yielded average Good coverage values of at least 0.9 at a depth of 1000, and all but 3 yielded an average of at least 0.8.


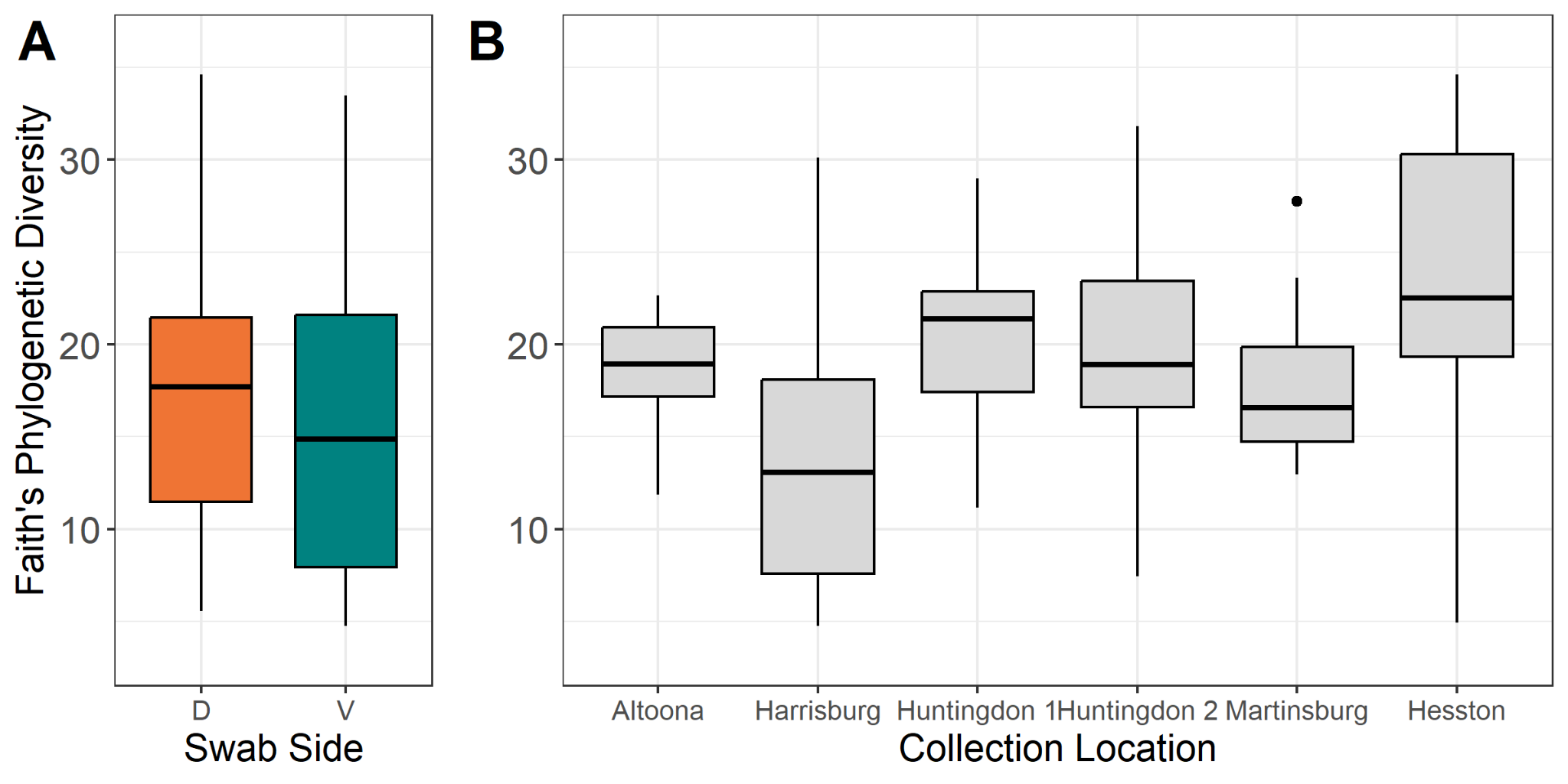


**Figure S2.** (A) Alpha diversity comparisons of bacterial ASVs on dorsal versus ventral cutaneous swabs. (B) Alpha diversity comparisons of bacterial ASVs by sampling location. Boxplots display the distribution of alpha diversity (Faith’s Phylogenetic Distance) across sampling sites. Points beyond the whiskers denote statistical outliers. This comparison highlights site-specific variation in cutaneous microbial richness. There is no statistical difference in ASV richness between dorsal and ventral swabs (Kruskal-Wallis, p = 0.125). There is a statistical difference in ASV richness between sampling sites (Kruskal-Wallis, p < 0.001). When pairwise comparisons among locations were evaluated using Kruskal-Wallis tests, the following were significant: Harrisburg vs. Hesston (p < 0.001), Harrisburg vs. Huntingdon 1 (p = 0.003), Harrisburg vs. Martinsburg (p = 0.005), Hesston vs. Martinsburg (p = 0.010).


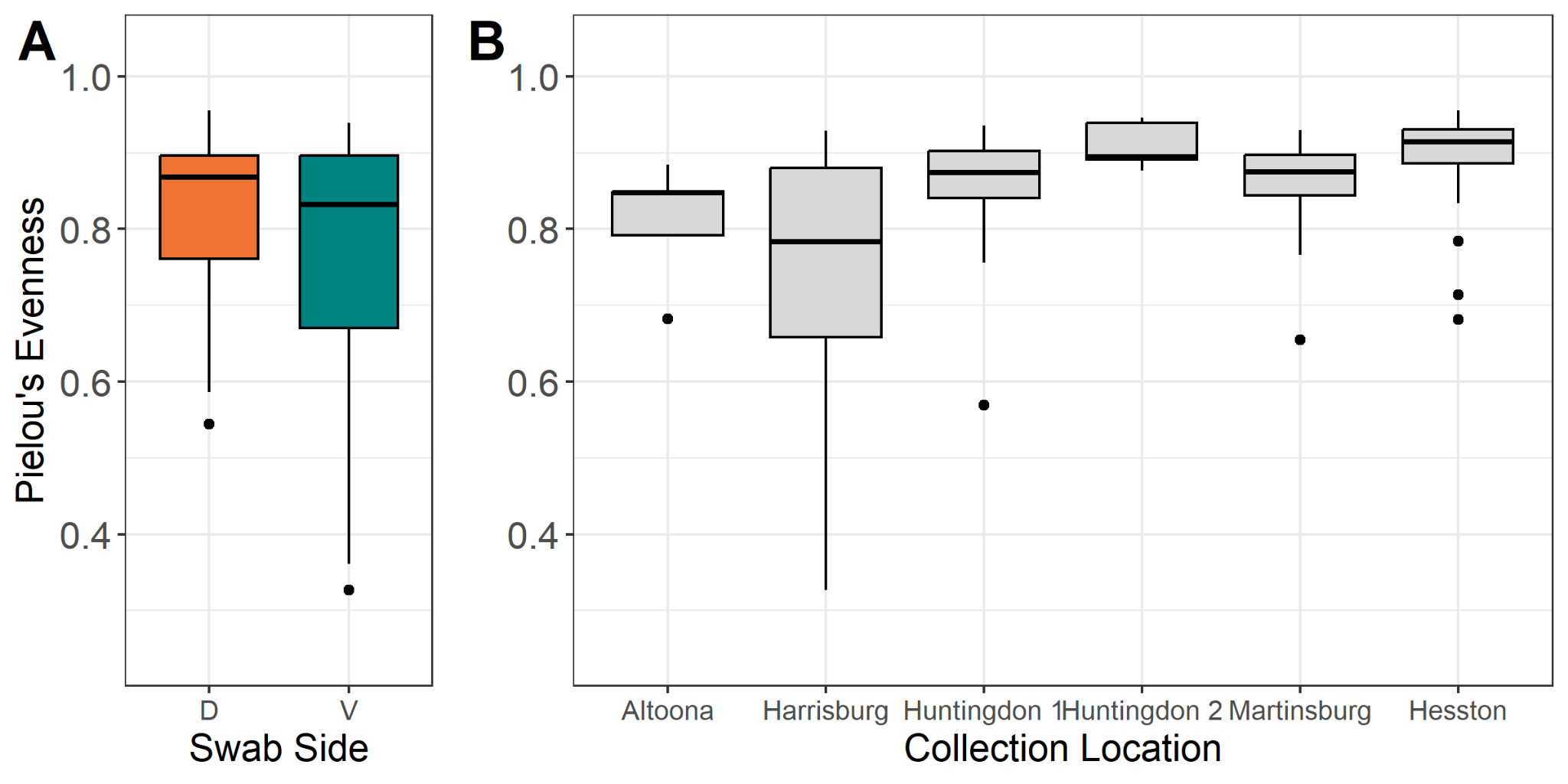


**Figure S3.** (A) Alpha diversity comparisons of bacterial ASVs on dorsal versus ventral cutaneous swabs. (B) Alpha diversity comparisons of bacterial ASVs by sampling location. Boxplots display the distribution of alpha diversity (Pielou’s evenness) across sampling sites. Points beyond the whiskers denote statistical outliers. This comparison highlights site-specific variation in cutaneous microbial richness. There is no statistical difference in ASV richness between dorsal and ventral swabs (Kruskal-Wallis, p = 0.139). There is a statistical difference in ASV richness between sampling sites (Kruskal-Wallis, p < 0.001). When pairwise comparisons among locations were evaluated using Kruskal-Wallis tests, the following were significant: Harrisburg vs. Hesston (p < 0.001), Harrisburg vs. Huntingdon 1 (p = 0.044), Harrisburg vs. Huntingdon 2 (p = 0.006), Harrisburg vs. Martinsburg (p = 0.004), Hesston vs. Altoona (p = 0.037), Hesston vs. Martinsburg (p = 0.026), Huntingdon 2 vs. Altoona (p = 0.016).


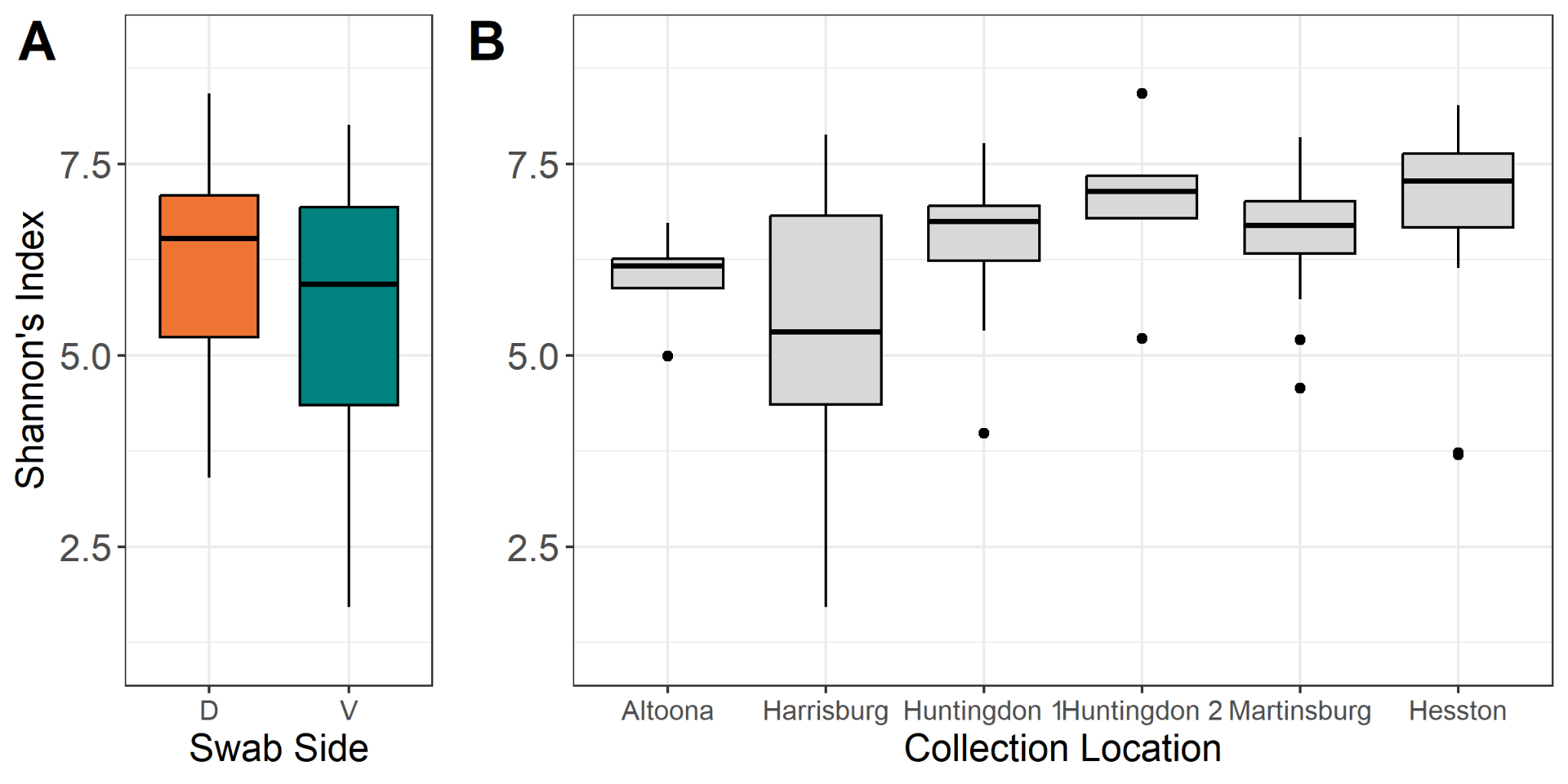


**Figure S4.** (A) Alpha diversity comparisons of bacterial ASVs on dorsal versus ventral cutaneous swabs. (B) Alpha diversity comparisons of bacterial ASVs by sampling location. Boxplots display the distribution of alpha diversity (Shannon’s index) across sampling sites. Points beyond the whiskers denote statistical outliers. This comparison highlights site-specific variation in cutaneous microbial richness. There is no statistical difference in ASV richness between dorsal and ventral swabs (Kruskal-Wallis, p = 0.057). There is a statistical difference in ASV richness between sampling sites (Kruskal-Wallis, p < 0.001). When pairwise comparisons among locations were evaluated using Kruskal-Wallis tests, the following were significant: Harrisburg vs. Hesston (p < 0.001), Harrisburg vs. Huntingdon 1 (p = 0.034), Harrisburg vs. Huntingdon 2 (p = 0.029), Harrisburg vs. Martinsburg (p = 0.003), Hesston vs. Altoona (p = 0.025).


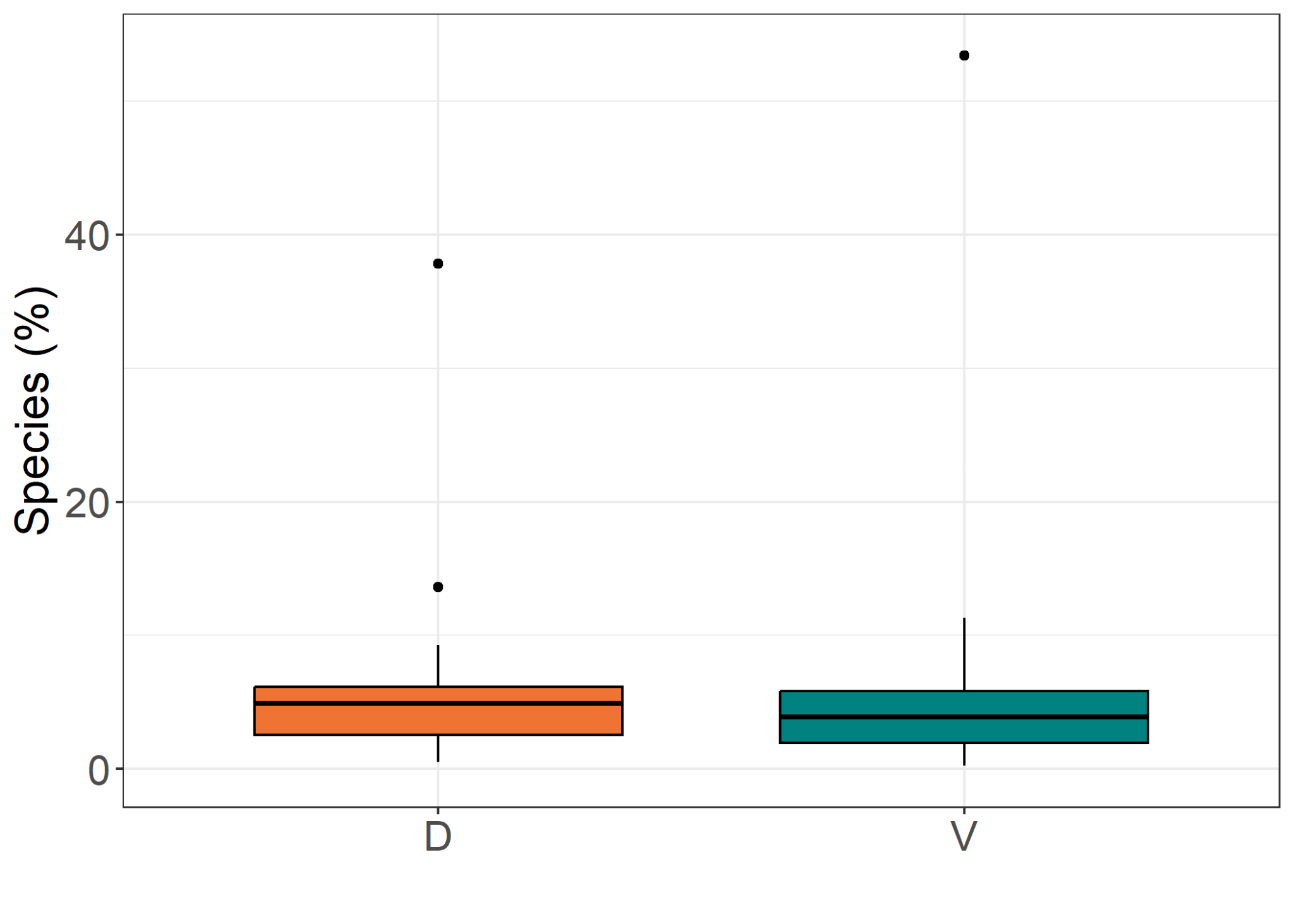


**Figure S5.** Boxplot of the percent of sequences remaining after all filtering in this study that could be identified to species level using a Naive Bayes classifier with 515F/806R sequences from the Silva 138.2 database.


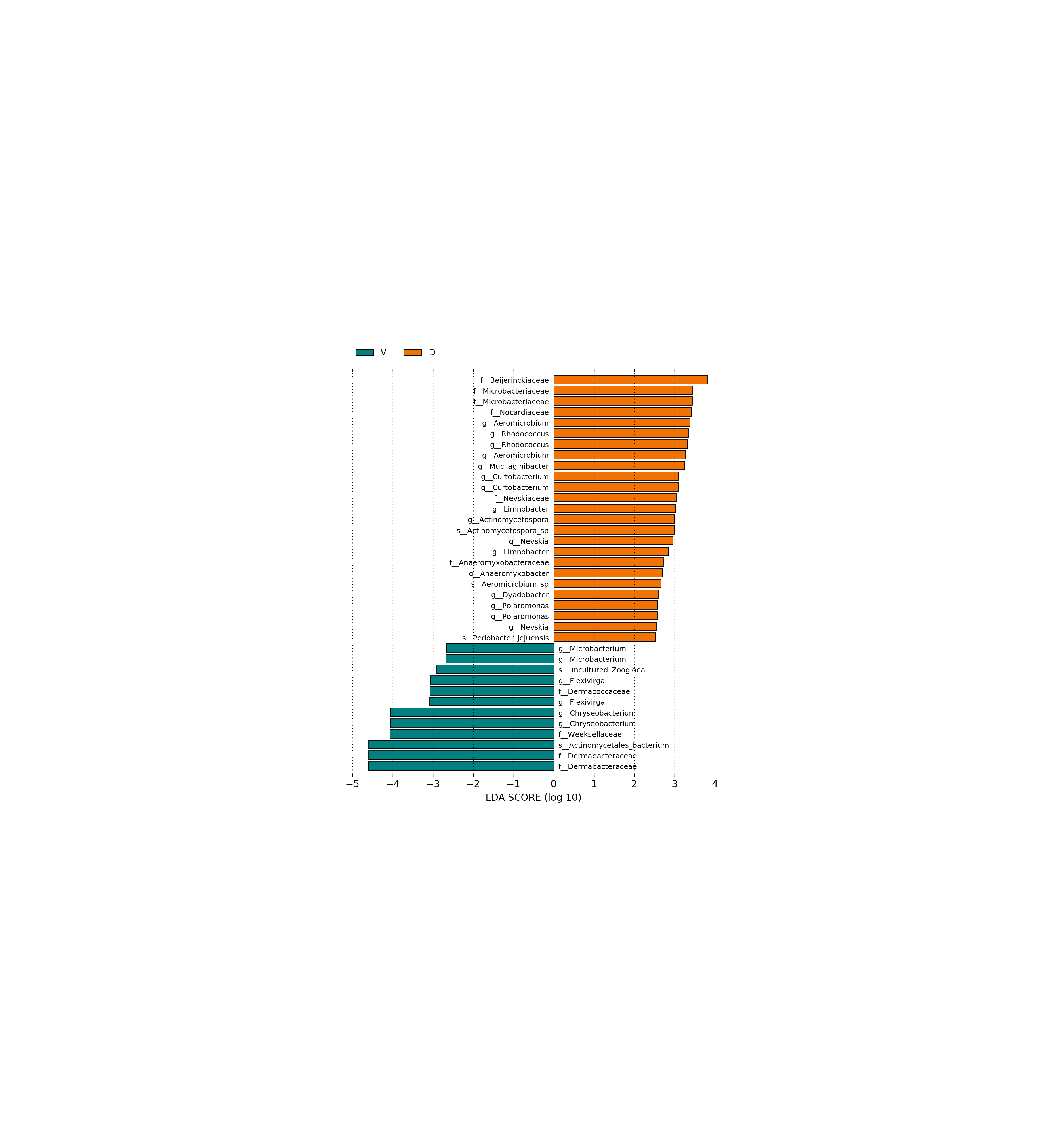


**Figure S6.** LEfSe plot of bacterial taxa differentially abundant between D (dorsal, orange) and V (ventral, teal) cutaneous microbiomes from toads. A Linear Discriminant Analysis (LDA) score (log10) of threshold of 2.5 was applied to identify differentially abundant features with at least family level taxonomy identified. Positive LDA scores denote bacterial taxa enriched in dorsal microbiomes, while negative LDA scores denote taxa enriched in ventral microbiomes.


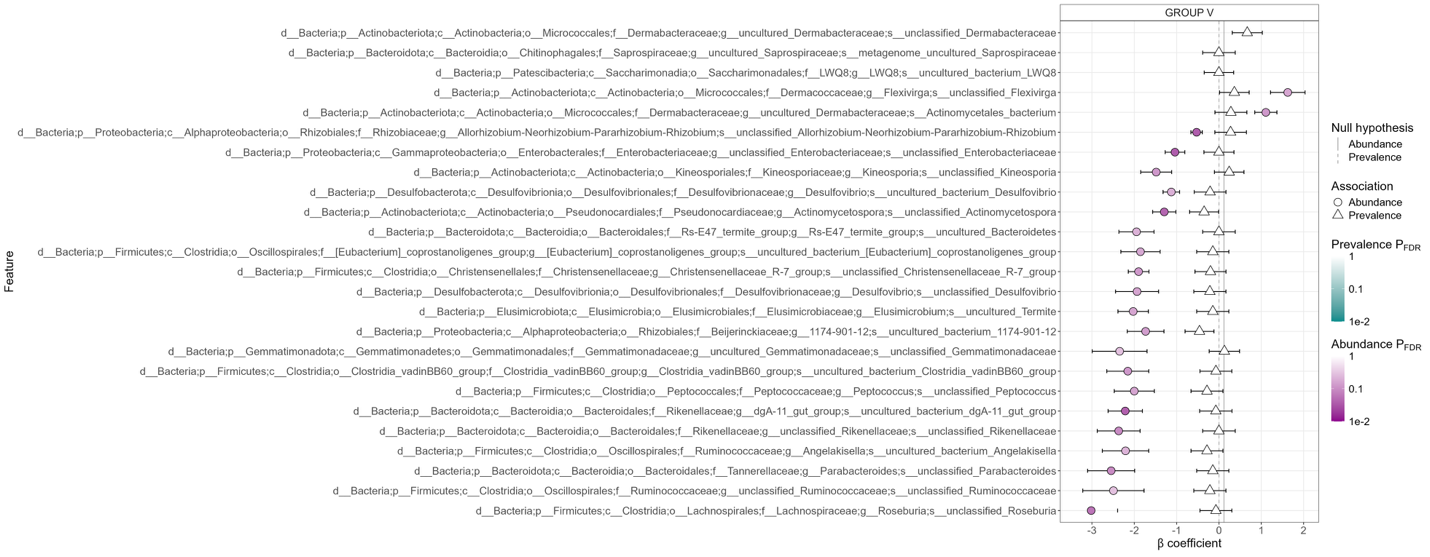


**Figure S7.** MaAsLin3 was used to identify taxonomic features that differ significantly between dorsal (left of midline) and ventral (right of midline) skin microbiomes. Triangles denote prevalence (proportion of samples in which a taxon was detected) and circles denote relative abundance for each significantly associated taxon, stratified by body region. Effect sizes represent MaAsLin3 model coefficients after adjustment for multiple testing (false discovery rate–corrected q values). This plot highlights body-region–specific structuring of the toad cutaneous microbiome, with distinct microbial assemblages characterizing dorsal versus ventral skin habitats.


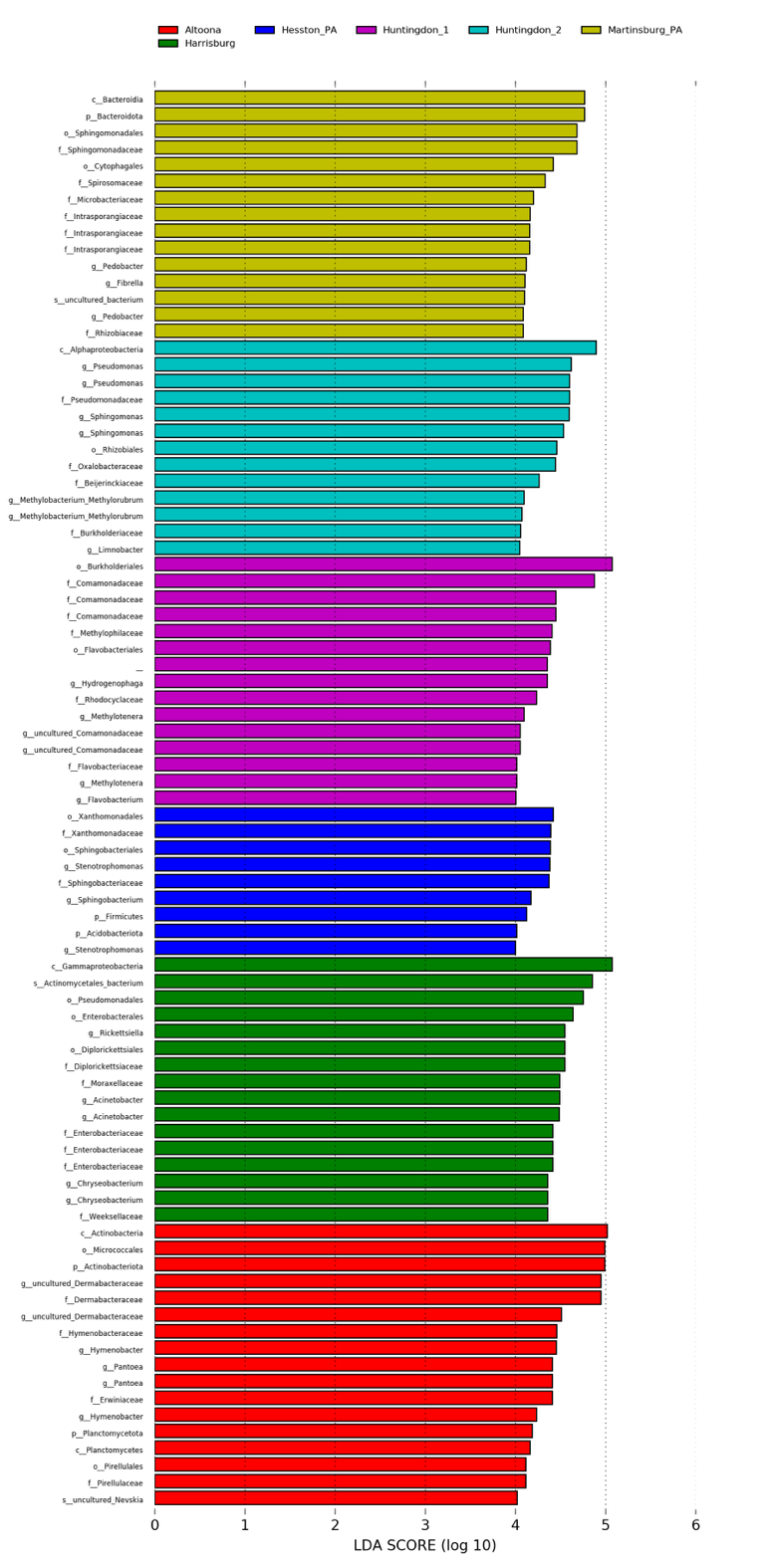


**Figure S8.** LEfSe plot of bacterial taxa differentially abundant in cutaneous microbiomes from toads collected from different sampling sites in Pennsylvania. A Linear Discriminant Analysis (LDA) score (log10) threshold of 4 was applied to identify differentially abundant features. Bars indicated bacterial taxa enriched in sampling sites from which toads were collected: Red=Altoona, Green=Harrisburg, Dark blue=Hesston, Teal and Purple=Huntingdon, Yellow=Martinsburg.

**Table S1.** LEfSe LDA scores and p-values (Kruskal-Wallis test) for bacterial taxa enriched in D (dorsal) and V (ventral) cutaneous microbiomes of toads.

| **Taxa** | **LDA score** | **p-val** | **Swab side** |
| --- | --- | --- | --- |
| d__Bacteria.p__Pseudomonadota.c__Alphaproteobacteria.o__Hyphomicrobiales.f__Beijerinckiaceae | 3.82318 | 0.045931 | D |
| d__Bacteria.p__Actinomycetota.c__Actinobacteria.o__Micrococcales.f__Microbacteriaceae | 3.438156 | 0.015958 | D |
| d__Bacteria.p__Actinomycetota.c__Actinobacteria.o__Micrococcales.f__Microbacteriaceae | 3.438156 | 0.015958 | D |
| d__Bacteria.p__Actinomycetota.c__Actinobacteria.o__Mycobacteriales.f__Nocardiaceae | 3.414603 | 0.013143 | D |
| d__Bacteria.p__Actinomycetota.c__Actinobacteria.o__Propionibacteriales.f__Nocardioidaceae.g__Aeromicrobium | 3.383922 | 0.013379 | D |
| d__Bacteria.p__Actinomycetota.c__Actinobacteria.o__Mycobacteriales.f__Nocardiaceae.g__Rhodococcus | 3.337579 | 0.002822 | D |
| d__Bacteria.p__Actinomycetota.c__Actinobacteria.o__Mycobacteriales.f__Nocardiaceae.g__Rhodococcus | 3.3194 | 0.001881 | D |
| d__Bacteria.p__Actinomycetota.c__Actinobacteria.o__Propionibacteriales.f__Nocardioidaceae.g__Aeromicrobium | 3.275357 | 0.040492 | D |
| d__Bacteria.p__Bacteroidota.c__Bacteroidia.o__Sphingobacteriales.f__Sphingobacteriaceae.g__Mucilaginibacter | 3.250488 | 0.040623 | D |
| d__Bacteria.p__Actinomycetota.c__Actinobacteria.o__Micrococcales.f__Microbacteriaceae.g__Curtobacterium | 3.099289 | 0.0105 | D |
| d__Bacteria.p__Actinomycetota.c__Actinobacteria.o__Micrococcales.f__Microbacteriaceae.g__Curtobacterium | 3.099289 | 0.0105 | D |
| d__Bacteria.p__Pseudomonadota.c__Gammaproteobacteria.o__Salinisphaerales.f__Nevskiaceae | 3.041415 | 0.007761 | D |
| d__Bacteria.p__Pseudomonadota.c__Gammaproteobacteria.o__Burkholderiales.f__Burkholderiaceae.g__Limnobacter | 3.032467 | 0.011229 | D |
| d__Bacteria.p__Actinomycetota.c__Actinobacteria.o__Pseudonocardiales.f__Pseudonocardiaceae.g__Actinomycetospora | 2.996633 | 0.03215 | D |
| d__Bacteria.p__Actinomycetota.c__Actinobacteria.o__Pseudonocardiales.f__Pseudonocardiaceae.g__Actinomycetospora.s__Actinomycetospora_sp | 2.993981 | 0.025983 | D |
| d__Bacteria.p__Pseudomonadota.c__Gammaproteobacteria.o__Salinisphaerales.f__Nevskiaceae.g__Nevskia | 2.960948 | 0.003843 | D |
| d__Bacteria.p__Pseudomonadota.c__Gammaproteobacteria.o__Burkholderiales.f__Burkholderiaceae.g__Limnobacter | 2.843651 | 0.014059 | D |
| d__Bacteria.p__Myxococcota.c__Myxococcia.o__Myxococcales.f__Anaeromyxobacteraceae | 2.717505 | 0.012529 | D |
| d__Bacteria.p__Myxococcota.c__Myxococcia.o__Myxococcales.f__Anaeromyxobacteraceae.g__Anaeromyxobacter | 2.696544 | 0.012529 | D |
| d__Bacteria.p__Actinomycetota.c__Actinobacteria.o__Propionibacteriales.f__Nocardioidaceae.g__Aeromicrobium.s__Aeromicrobium_sp | 2.660705 | 0.009059 | D |
| d__Bacteria.p__Bacteroidota.c__Bacteroidia.o__Cytophagales.f__Spirosomataceae.g__Dyadobacter | 2.591586 | 0.015488 | D |
| d__Bacteria.p__Pseudomonadota.c__Gammaproteobacteria.o__Burkholderiales.f__Comamonadaceae.g__Polaromonas | 2.574209 | 0.003158 | D |
| d__Bacteria.p__Pseudomonadota.c__Gammaproteobacteria.o__Burkholderiales.f__Comamonadaceae.g__Polaromonas | 2.56582 | 0.004622 | D |
| d__Bacteria.p__Pseudomonadota.c__Gammaproteobacteria.o__Salinisphaerales.f__Nevskiaceae.g__Nevskia | 2.548337 | 0.00358 | D |
| d__Bacteria.p__Bacteroidota.c__Bacteroidia.o__Sphingobacteriales.f__Sphingobacteriaceae.g__Pedobacter.s__Pedobacter_jejuensis | 2.52576 | 0.025778 | D |
| d__Bacteria.p__Actinomycetota.c__Actinobacteria.o__Micrococcales.f__Dermabacteraceae | 4.602482 | 0.001128 | V |
| d__Bacteria.p__Actinomycetota.c__Actinobacteria.o__Micrococcales.f__Dermabacteraceae | 4.597295 | 0.001298 | V |
| d__Bacteria.p__Actinomycetota.c__Actinobacteria.o__Micrococcales.f__Dermabacteraceae.g__Incertae_Sedis.s__Actinomycetales_bacterium | 4.597295 | 0.001298 | V |
| d__Bacteria.p__Bacteroidota.c__Bacteroidia.o__Flavobacteriales.f__Weeksellaceae | 4.065943 | 0.038461 | V |
| d__Bacteria.p__Bacteroidota.c__Bacteroidia.o__Flavobacteriales.f__Weeksellaceae.g__Chryseobacterium | 4.063766 | 0.039983 | V |
| d__Bacteria.p__Bacteroidota.c__Bacteroidia.o__Flavobacteriales.f__Weeksellaceae.g__Chryseobacterium | 4.052668 | 0.043596 | V |
| d__Bacteria.p__Actinomycetota.c__Actinobacteria.o__Micrococcales.f__Dermacoccaceae.g__Flexivirga | 3.082718 | 0.037364 | V |
| d__Bacteria.p__Actinomycetota.c__Actinobacteria.o__Micrococcales.f__Dermacoccaceae | 3.078421 | 0.022344 | V |
| d__Bacteria.p__Actinomycetota.c__Actinobacteria.o__Micrococcales.f__Dermacoccaceae.g__Flexivirga | 3.071272 | 0.037364 | V |
| d__Bacteria.p__Pseudomonadota.c__Gammaproteobacteria.o__Burkholderiales.f__Rhodocyclaceae.g__Zoogloea.s__uncultured_Zoogloea | 2.90639 | 0.046241 | V |
| d__Bacteria.p__Actinomycetota.c__Actinobacteria.o__Micrococcales.f__Microbacteriaceae.g__Microbacterium | 2.675736 | 0.001416 | V |

**Table S2.** PICRUSt2 p-values (Kruskal-Wallis test) for predicted functional pathways in V (ventral) and D (dorsal) skin microbiomes

| **Gene Pathways** | **LDA score** | **p-values** | **Swab side** |
| --- | --- | --- | --- |
| Metabolism.Xenobiotics_biodegradation_and_metabolism | 2.852085 | 0.032694 | D |
| Metabolism.Metabolism_of_cofactors_and_vitamins.Porphyrin_metabolism__PATH_ko00860_ | 2.481052 | 0.001014 | D |
| Cellular_Processes.Cellular_community___prokaryotes.Biofilm_formation___Escherichia_coli__PATH_ko02026_ | 2.320595 | 0.032694 | D |
| Metabolism.Amino_acid_metabolism.Arginine_and_proline_metabolism__PATH_ko00330_ | 2.252609 | 0.005252 | D |
| Cellular_Processes.Cell_growth_and_death.Cell_cycle___Caulobacter__PATH_ko04112_ | 2.115499 | 0.024341 | D |
| Environmental_Information_Processing.Signal_transduction.mTOR_signaling_pathway__PATH_ko04150_ | 2.061096 | 0.02098 | D |
| Genetic_Information_Processing | 3.236245 | 0.02409 | V |
| Genetic_Information_Processing.Replication_and_repair | 2.852425 | 0.011104 | V |
| Genetic_Information_Processing.Translation | 2.823125 | 0.022627 | V |
| Genetic_Information_Processing.Translation.Ribosome__PATH_ko03010_ | 2.68763 | 0.024851 | V |
| Genetic_Information_Processing.Folding__sorting_and_degradation | 2.47117 | 0.040179 | V |
| Metabolism.Nucleotide_metabolism | 2.44972 | 0.033685 | V |
| Metabolism.Energy_metabolism.Oxidative_phosphorylation__PATH_ko00190_ | 2.38445 | 0.040569 | V |
| Metabolism.Carbohydrate_metabolism.Citrate_cycle__TCA_cycle___PATH_ko00020_ | 2.361781 | 0.020798 | V |
| Genetic_Information_Processing.Replication_and_repair.Homologous_recombination__PATH_ko03440_ | 2.308608 | 0.005866 | V |
| Metabolism.Amino_acid_metabolism.Cysteine_and_methionine_metabolism__PATH_ko00270_ | 2.305615 | 6.84E-05 | V |
| Metabolism.Amino_acid_metabolism.Phenylalanine__tyrosine_and_tryptophan_biosynthesis__PATH_ko00400_ | 2.280319 | 0.000447 | V |
| Metabolism.Nucleotide_metabolism.Purine_metabolism__PATH_ko00230_ | 2.277473 | 0.044226 | V |
| Genetic_Information_Processing.Replication_and_repair.DNA_replication__PATH_ko03030_ | 2.226118 | 0.002969 | V |
| Genetic_Information_Processing.Translation.Aminoacyl_tRNA_biosynthesis__PATH_ko00970_ | 2.210936 | 0.031413 | V |
| Metabolism.Metabolism_of_cofactors_and_vitamins.One_carbon_pool_by_folate__PATH_ko00670_ | 2.19207 | 0.020798 | V |
| Metabolism.Metabolism_of_cofactors_and_vitamins.Nicotinate_and_nicotinamide_metabolism__PATH_ko00760_ | 2.187632 | 0.000986 | V |
| Metabolism.Amino_acid_metabolism.Histidine_metabolism__PATH_ko00340_ | 2.163861 | 0.002438 | V |
| Genetic_Information_Processing.Replication_and_repair.Mismatch_repair__PATH_ko03430_ | 2.134022 | 0.003379 | V |
| Genetic_Information_Processing.Replication_and_repair.Nucleotide_excision_repair__PATH_ko03420_ | 2.114195 | 0.030175 | V |
| Cellular_Processes.Transport_and_catabolism.Peroxisome__PATH_ko04146_ | 2.076871 | 0.002471 | V |
| Metabolism.Glycan_biosynthesis_and_metabolism.Peptidoglycan_biosynthesis__PATH_ko00550_ | 2.037354 | 2.73E-02 | V |

**Table S3.** Taxa Contributions of Enriched Functional Gene Pathways in Dorsal Microbiomes for Xenobiotic Biodegradation and Porphyrin Metabolism

|  | **(Mean %)** | **Standard Error** | **Median** | **Interquartile Range** |
| --- | --- | --- | --- | --- |
| **Xenobiotics Biodegradation** | | | | |
| d__Bacteria;p__Pseudomonadota;c__Gammaproteobacteria;o__Pseudomonadales;f__Pseudomonadaceae;g__Pseudomonas;s__unclassified_Pseudomonas | 10.86 | 1.121 | 7.866 | 14.652 |
| d__Bacteria;p__Pseudomonadota;c__Alphaproteobacteria;o__Sphingomonadales;f__Sphingomonadaceae;g__Sphingomonas;s__unclassified_Sphingomonas | 9.116 | 0.862 | 8.763 | 13.106 |
| d__Bacteria;p__Pseudomonadota;c__Gammaproteobacteria;o__Pseudomonadales;f__Moraxellaceae;g__Acinetobacter;s__unclassified_Acinetobacter | 4.352 | 0.919 | 0.347 | 4.338 |
| d__Bacteria;p__Pseudomonadota;c__Alphaproteobacteria;o__Hyphomicrobiales;f__Rhizobiaceae;g__unclassified_Rhizobiaceae;s__unclassified_Rhizobiaceae | 4.11 | 0.376 | 3.711 | 4.067 |
| d__Bacteria;p__Actinomycetota;c__Actinobacteria;o__Micrococcales;f__Dermabacteraceae;g__Incertae_Sedis;s__Actinomycetales_bacterium | 4.096 | 0.719 | 1.334 | 5.952 |
| d__Bacteria;p__Pseudomonadota;c__Gammaproteobacteria;o__Burkholderiales;f__Comamonadaceae;g__unclassified_Comamonadaceae;s__unclassified_Comamonadaceae | 3.902 | 0.492 | 3.047 | 4.231 |
| d__Bacteria;p__Pseudomonadota;c__Gammaproteobacteria;o__Enterobacterales;f__Enterobacteriaceae;g__unclassified_Enterobacteriaceae;s__unclassified_Enterobacteriaceae | 3.82 | 0.786 | 0.084 | 6.397 |
| d__Bacteria;p__Pseudomonadota;c__Alphaproteobacteria;o__Hyphomicrobiales;f__Beijerinckiaceae;g__Methylobacterium;s__unclassified_Methylobacterium | 3.343 | 0.419 | 2.618 | 4.77 |
| d__Bacteria;p__Bacteroidota;c__Bacteroidia;o__Flavobacteriales;f__Weeksellaceae;g__Chryseobacterium;s__unclassified_Chryseobacterium | 2.705 | 0.376 | 2.008 | 2.906 |
| d__Bacteria;p__Pseudomonadota;c__Gammaproteobacteria;o__Burkholderiales;f__Oxalobacteraceae;g__Massilia;s__unclassified_Massilia | 2.415 | 0.221 | 1.905 | 2.77 |
| **Porphyrin Metabolism** | | | | |
| d__Bacteria;p__Pseudomonadota;c__Gammaproteobacteria;o__Pseudomonadales;f__Pseudomonadaceae;g__Pseudomonas;s__unclassified_Pseudomonas | 11.224 | 1.159 | 8.036 | 16.129 |
| d__Bacteria;p__Pseudomonadota;c__Alphaproteobacteria;o__Sphingomonadales;f__Sphingomonadaceae;g__Sphingomonas;s__unclassified_Sphingomonas | 9.392 | 0.91 | 8.7 | 13.79 |
| d__Bacteria;p__Pseudomonadota;c__Gammaproteobacteria;o__Enterobacterales;f__Enterobacteriaceae;g__unclassified_Enterobacteriaceae;s__unclassified_Enterobacteriaceae | 5.043 | 1.059 | 0.064 | 7.77 |
| d__Bacteria;p__Pseudomonadota;c__Alphaproteobacteria;o__Hyphomicrobiales;f__Beijerinckiaceae;g__Methylobacterium;s__unclassified_Methylobacterium | 4.997 | 0.605 | 3.874 | 7.157 |
| d__Bacteria;p__Actinomycetota;c__Actinobacteria;o__Micrococcales;f__Dermabacteraceae;g__Incertae_Sedis;s__Actinomycetales_bacterium | 3.912 | 0.7 | 1.058 | 5.762 |
| d__Bacteria;p__Pseudomonadota;c__Gammaproteobacteria;o__Rickettsiellales;f__Rickettsiellaceae;g__Rickettsiella;s__uncultured_Rickettsiella | 3.613 | 1.236 | 0 | 0.008 |
| d__Bacteria;p__Pseudomonadota;c__Alphaproteobacteria;o__Hyphomicrobiales;f__Rhizobiaceae;g__unclassified_Rhizobiaceae;s__unclassified_Rhizobiaceae | 3.444 | 0.31 | 3.013 | 3.556 |
| d__Bacteria;p__Pseudomonadota;c__Gammaproteobacteria;o__Burkholderiales;f__Comamonadaceae;g__unclassified_Comamonadaceae;s__unclassified_Comamonadaceae | 2.961 | 0.392 | 1.922 | 3.359 |
| d__Bacteria;p__Bacteroidota;c__Bacteroidia;o__Flavobacteriales;f__Weeksellaceae;g__Chryseobacterium;s__unclassified_Chryseobacterium | 2.904 | 0.415 | 1.854 | 3.029 |
| d__Bacteria;p__Pseudomonadota;c__Gammaproteobacteria;o__Burkholderiales;f__Oxalobacteraceae;g__Massilia;s__unclassified_Massilia | 2.05 | 0.199 | 1.577 | 2.234 |
